# Standardization of Methodology of Light-to-Heat Conversion Efficiency Determination for Colloidal Nanoheaters

**DOI:** 10.1021/acsami.1c12409

**Published:** 2021-09-09

**Authors:** Agnieszka Paściak, Aleksandra Pilch-Wróbel, Łukasz Marciniak, P. James Schuck, Artur Bednarkiewicz

**Affiliations:** † Institute of Low Temperature and Structure Research, Polish Academy of Sciences, Okólna 2, 50-422 Wrocław, Poland; ‡ Department of Mechanical Engineering, Columbia University, New York, New York 10027, United States

**Keywords:** photothermal conversion efficiency, photothermal therapy, gold nanoparticles, standardization, nanoheaters

## Abstract

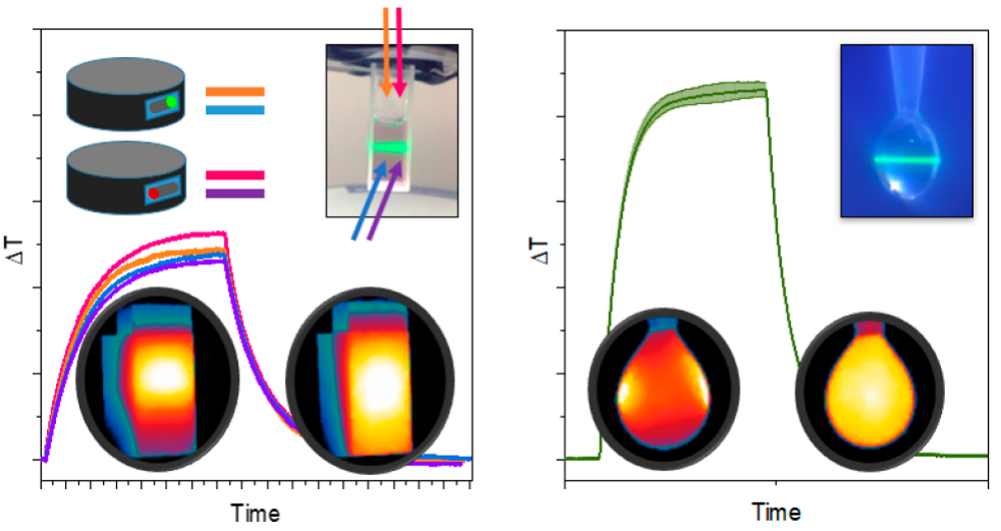

Localized photothermal therapy (PTT) has been demonstrated to be a promising method of combating cancer, that additionally synergistically enhances other treatment modalities such as photodynamic therapy or chemotherapy. PTT exploits nanoparticles (called nanoheaters), that upon proper biofunctionalization may target cancerous tissues, and under light stimulation may convert the energy of photons to heat, leading to local overheating and treatment of cancerous cells. Despite extensive work, there is, however, no agreement on how to accurately and quantitatively compare light-to-heat conversion efficiency (η_*Q*_) and rank the nanoheating performances of various groups of nanomaterials. This disagreement is highly problematic because the obtained η_*Q*_ values, measured with various methods, differ significantly for similar nanomaterials. In this work, we experimentally review existing optical setups, methods, and physical models used to evaluate η_*Q*_. In order to draw a binding conclusion, we cross-check and critically evaluate the same Au@SiO_2_ sample in various experimental conditions. This critical study let us additionally compare and understand the influence of the other experimental factors, such as stirring, data recording and analysis, and assumptions on the effective mass of the system, in order to determine η_*Q*_ in a most straightforward and reproducible way. Our goal is therefore to contribute to the understanding, standardization, and reliable evaluation of η_*Q*_ measurements, aiming to accurately rank various nanoheater platforms.

## 1 Introduction

Photothermal therapy (PTT), alternatively named hyperthermia (HT), has been proposed to become adjuvant cancer treatment^1^ to other well-known therapeutic methods such as photodynamic therapy^2^ or chemotherapy.^3^ This remote, minimally invasive (due to the need to inject exogenous functional nanomaterials) technique can not only combat cancer by itself, but has shown synergistic enhancement of therapeutic effects as compared to singular treatments. It is widely accepted that photothermal therapy exploits natural negative susceptibility of cancerous tissues to increased temperatures, as compared to normal tissues.^4^ This can be explained on the basis of biochemistry and biophysics at the cellular level; for example, the research conducted on hepatoma cells has shown that at increased temperature (43 °C) in aerobic conditions, cellular respiration^5^ and also protein synthesis^6^ have been inhibited. Moreover, the difference in lability of cell membranes (surface or lysosomal) in healthy and cancerous cells has been raised.^5,6^

There are numerous ways cancerous tissues can be overheated above the physiological level (typically 39–45 °C).^7^ Whole-body hyperthermia is highly exhausting for patients, and thus has been replaced with localized heat deposition methods exploiting hyperthermic nanoparticles (HTNPs). Such an approach requires further extensive studies and versatile chemical, physical, biochemical, and biological examination and evaluation of performance of various nanoparticles before they can be accepted for medical use in vivo. Among these features the chemistry (e.g., simple and cost-effective synthesis and the stability of colloidal NPs), biosafety (e.g., circulation time, deposition of HTNPs within organs and their clearance from the body,^8^ lack of inherent primary or secondary toxicity), functionality (e.g., simple biofunctionalization and selective targeting of the cancerous tissues, heating and thermometry within a single nanoparticle for feedback controlled HT), and efficiency (i.e., light-to-heat conversion efficiency (η_*Q*_) of HTNPs, and lethal light dose–response) are the most important parameters and methods to understand, optimize, or verify.

So far, numerous photothermal nanoheaters that fulfill most of the above-mentioned requirements have been proposed,^9^ and their fundamental properties as well as feasibility for PTT has been demonstrated. In particular, these HTNPs include gold nanoparticles (AuNPs) in different shapes (nanospheres,^10^ nanorods,^11^ nanostars,^12^ and others), as well as lanthanide doped nanoparticles,^13,14^ quantum dots,^15^ organic dyes,^16^ transition metal dichalcogenides,^17^ polymers,^18,19^ and carbon-,^20,21^ iron-,^22^ and titanium-based^23^ nanomaterials. In addition to these materials, semiconductor^24,25^ and dielectric^26,27^ materials have also shown very promising properties of converting light into heat, and even higher absorption coefficients were found as compared to plasmonic ones. The selection of the most appropriate NPs for PTT is not trivial because many features such as particle size, irradiation wavelength and time, η_*Q*_, absorption cross-section, and shell surface are equally important for the potential adoption of particular NPs for PTT therapy. A small particle size and narrow size distribution is recommended to enable HTNPs to pass through the vascular system. For systemic administration, particle size should be smaller than 200 nm,^28^ although there are studies that claimed that size under 50 nm provides fewer side effects.^29^ Second, the specific interaction and accumulation of HTNPs in cancerous tissues require appropriate antibody-conjugation of the NPs,^30^ while the nanoparticles should exhibit low inherent cytotoxicity, good biocompatibility and the possibility of fast clearance from the body after therapy. Third, the photoexcitation light wavelength (specifically for light induced heating) must fit one of the optical biological windows^31^ to enable deep light penetration through the skin without excessive absorption and scattering, as well as without excessive heating and damaging of tissues by the light itself. Last, but not least, high η_*Q*_ values are required likewise high light-absorption cross section of given HTNPs, owing to restricted maximum permissible exposure (MPE) dose on the skin surface (according to the IEC-60825-1 and ANSI Z136.1 standards)^32,33^ as well as unknown, and hypothetically low concentration of HTNPs in the targeted tissue.

**Figure 1:**
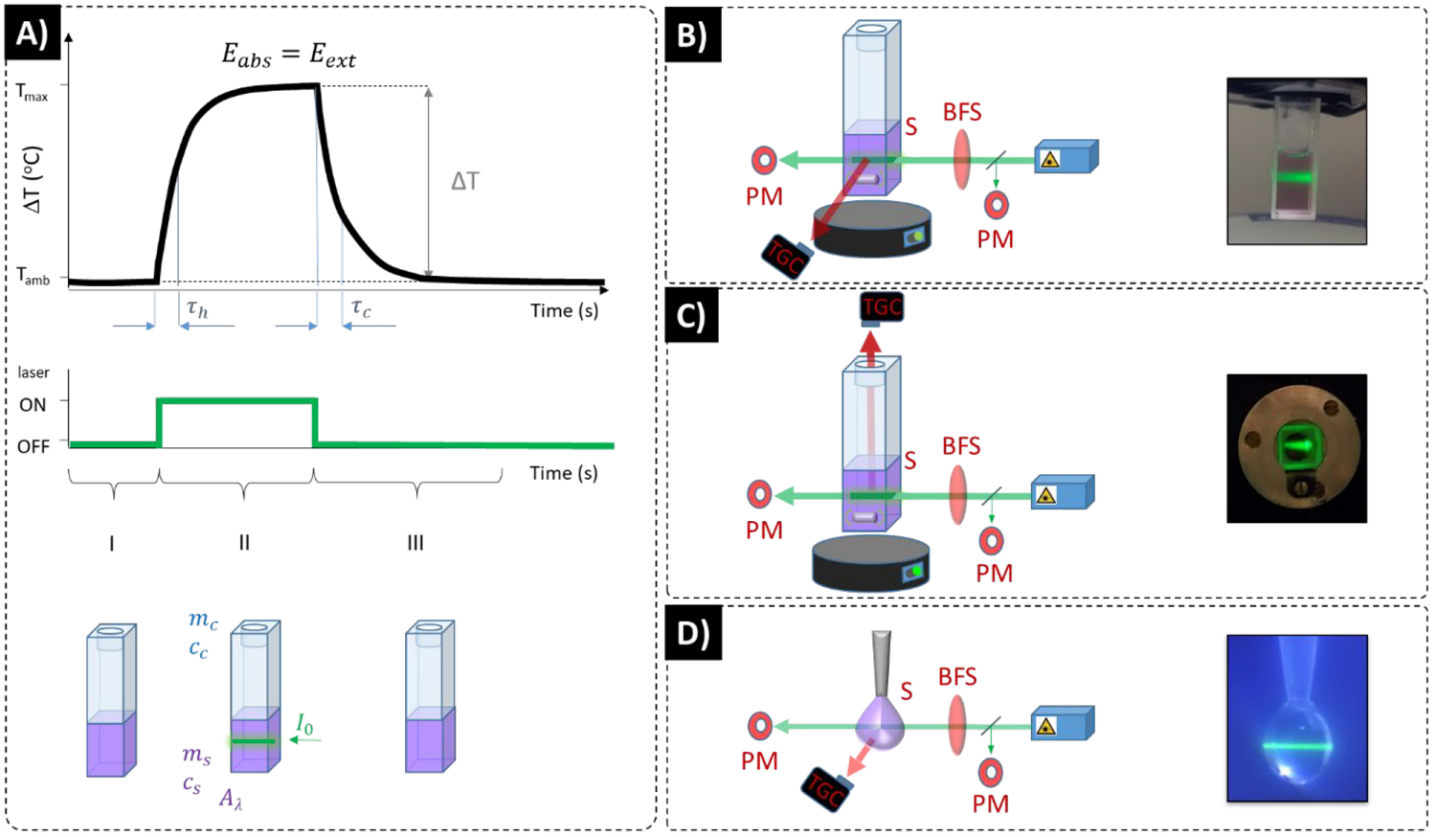
General scheme of methods and setups used to determine η_*Q*_. (A) Schematic presentation of the elements and methods used in heat balance physical model (bottom row), photostimulation (middle row), and the exemplary results of typical η_*Q*_ measurements (top row); τ_h_ and τ_c_ denote inverse of heating and cooling rates, respectively, at saturation stage *E*_abs_ = *E*_ext_, where *E*_abs_ and *E*_ext_ are absorbed and external energies, the Δ*T* is maximum temperature rise; *m*_*x*_ and *c*_*x*_ denote the mass and heat capacity of the cuvette (*x* = C) and of the sample (*x* = S), the sample is heated at stage II, while stabilizing at stage I and cooling down at stage III; schematic presentation and example photos of experimental setup variants: (B) Thermographic camera (TGC) records the temperature of side wall of the cuvette, (C) TGC records the temperature of colloidal nanoparticle solution directly from the top of the cuvette, (D) TGC records the temperature images of a small droplet of colloidal HTNPs. PM is optical power meter, BFS is a beam focusing system, S is a sample. Mild sample stirring may be applied in (B) and (C) setups.

The figure of merit for the PTT, which determines the feasibility of using a particular type of nanoparticles for HT and is comprehensively studied here, is light-to-heat conversion efficiency. However, due to the fact that in the literature on nanoheaters the efficiency of converting light into heat is not always determined,^9^ it is difficult to quantitatively compare them in this respect. There have been many attempts to determine η_*Q*_,^12,34−36^ but the main objection is that the results obtained by most of them depend on the measurement conditions. Notably, many reports can be found that significantly differ in the evaluated η_*Q*_ for the same or very similar materials. We, therefore, see an urgent need to standardize the measurement techniques and experimental factors, which may ultimately affect the quantitative evaluation of η_*Q*_ between materials. A standardized η_*Q*_ measurement and analysis will in consequence enable proper comparison and ranking of various materials in different laboratories. Hence, we have critically assessed various existing light-to-heat conversion physical models, as well as built various optical setups (Figure 1) and evaluated many experimental factors using exactly the same batch of Au@SiO_2_ NPs. We have chosen this HT nanomaterial as a prototypical example, because gold nanoparticles are one of the most common and intensively studied classes of HTNPs. The silica coating improves colloidal stability,^37^ enhances biocompatibility, stabilizes NPs shape and surface, and enhances thermal stability.^38^ Moreover, using NPs that are dispersible in water, which is a prerequisite for bioapplications, makes it easier to compare them with other agents and easier to further functionalize them.

It has been proposed and demonstrated that heat generated in nanoscale plasmonic particles may depend on the nanoparticle morphology, beam incidence orientation, and may display high nonuniformity around such singular objects or aggregates.^39^ In our work, however, we adopt a simplified, but more general approach, by treating the colloidal nanoparticle solutions as homogeneous materials without entering into their nanoscale nature, which seems to be adequate for numerous types of other nonplasmonic nanoheaters, such as lanthanide doped NPs, quantum dots, and carbon based nanomaterials.

Almost all of the currently existing models are based on the analysis of experimentally measured temperature response kinetics resulting from heating the sample by a continuous wave laser beam. At first, ambient temperature is recorded (phase I in Figure 1A) and is followed by optical stimulation, which due to light-to-heat conversion increases the temperature of the sample (phase II in Figure 1A). Ultimately, saturated temperature is reached, and after switching the photostimulation off, the sample spontaneously cools down back to ambient temperature (phase III in Figure 1A). These heating–cooling kinetic profiles are key for evaluating the capability and efficiency of the water dispersed colloidal nanoparticle heaters to increase local temperature under photostimulation.

The starting point of η_*Q*_ calculations for the majority of existing models is the heat balance equation (eq 1), which describes the heating of the nanoparticle solution by continuous wave lasers. When ultrashort pulses of the excitation are applied, the equation should be expanded to take into account the average pumping power based on the energy, pulse duration, and pulse frequencies. Nevertheless, the major aim of our manuscript is to quantitatively investigate and compare light-to-heat conversion efficiency of the same batch of the sample with various optical setups, sample holders, and models, aiming to develop a most convenient and reliable quantification methodology.
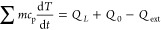
1

Σ*mc*_p_ is a sum of products of mass and heat capacities of all system components, is the rate of temperature increase, *Q*_ext_ is external heat flux, *Q*_O_ + *Q*_L_ is heat produced by converting the absorbed light into heat, by either solvent (*Q*_O_) or by the nanoparticles (*Q*_L_). The *Q*_L_ is determined with the following equation:
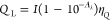
2where *I* is a laser power, *A*_λ_ is the absorbance at irradiation wavelength λ and is measured experimentally using Lambert–Beer’s law. In equilibrium conditions,  in eq 1 and η_*Q*_ can be calculated as^34^
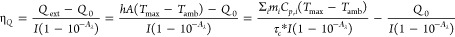
3

In this model, *Q*_ext_ could be computed from experimental cooling kinetics, where *h* is the heat transfer coefficient, *A* is the surface area for heat transfer to surroundings, *T*_amb_ is the temperature of surroundings, and *T* is the current temperature. In equilibrium conditions, the actual temperature equals steady-state temperature *T* = *T*_max_ = *T*_amb_ + Δ*T*. According to literature,^34^ these parameters could be approximated by a sum of products of mass and heat capacities of all system components and cooling time coefficient τ_*c*_. As we will discuss later, it is clear from eq 3 that there are a number of experimental factors which will ultimately affect the η_*Q*_ absolute value. For example, either the accuracy of τ_*c*_ determination, interpretation of which elements of the system should be considered in the sum of mass and heat capacity product Σ_*i*_*m*_*i*_*c*_*p,i*_, or the method of actual temperature determination (Figure 1B–D) are all not trivial and may significantly affect the final results. For example, the results from the simulation presented by Marin et al.^40^ differ from 63.8% to 91.4% for the same sample with different setup characterizing data. The τ_*c*_ is typically calculated as the exponential slope coefficient of the cooling part of heating–cooling kinetic profiles (Figure 1A). However, such an approach is not intuitive from the physics perspective, and it is noteworthy that the heating and the cooling time coefficients in all of our experiments differ. For instance, in the “top view” experiment (Figure 1C) with stirring, a cooling time coefficient was longer than the heating time coefficient. Although Richardson et al.^35^ approximated the heating time rate for the cooling part of the profile, we suppose that the time rates may vary due to different heat propagation mechanisms, for example, collective heating and cooling by heat diffusion. It was found out that cooling rate is affected strongly by the material from which the holder (adjacent to the sample during the experiment) was made. The heater should be more effective if it heats the given volume faster, thus the heater’s efficiency should be proven by the heating time rate.

As opposed to Roper’s model, Wang’s model^12^ is also derived from the heat balance model (eq 1), but is converted into a different form:
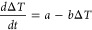
4where
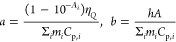
5

Based on that, the η_*Q*_ was calculated from equation:
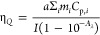
6where parameter *a* (K/s) describes how much of the incident power *I* (W/cm^2^) can be effectively stored by the system of heat capacity *C*_p_, that translates into how much the temperature changes per *unit time*. It must be underlined here that due to the different optical properties of nanomaterials at different wavelengths, η_*Q*_ depends on the excitation wavelength. Therefore, it is important to provide not only the η_Q_, but wavelength specific η_*Q*_ (λ), which also means the η_*Q*_ cannot be directly compared in similar materials unless the excitation wavelength and absorption coefficient (at least) or spectra are provided. The *a* can be calculated from the rising part of the heating–cooling kinetic profiles:
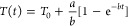
7

Theoretically, based on the assumption that the time constant of heating and cooling are identical^12,35^ and assuming that the mass and heat capacities of the measuring system components are taken into account in the same way, both models should lead to similar results. However, this assumption may be wrong because Wang’s model accounts for the mass of the system differently, that is, an effective mass of the measurement setup is considered instead of a simple sum of contributions from all the components. Moreover, as alluded to above, recent research shows that nanoparticles heat up more quickly than they cool down,^41^ which raises new questions for the characterization of nanoheaters. Since the development of these phenomenological models and experimental methods, whose similarities and differences were presented above, many different materials have been characterized for their suitability for PTT. Nevertheless, the variability of experimental setups, measurement conditions, and assumptions used for data acquisition and analysis, make the conducted studies almost impossible to directly compare. Large discrepancies in experimentally obtained η_*Q*_ values can be found in the scientific literature for similar nanoheaters, and cannot be unequivocally ascribed to the actual differences in nanoparticles used.

## 2 Results and Discussion

### 2.1 Light-to-Heat Conversion Efficiency: The Models

Because there is no agreement in the literature on which method of the η_*Q*_ determination is the most accurate, we were motivated to quantitatively compare the existing models and measurement setups. Not only did we evaluate the same batch of the sample, but also verified how variants of the experimental conditions (e.g., mechanical stirring or still colloid, side-wall or direct colloid temperature evaluation, the way the temperature is recorded, corrected, and analyzed, etc.) might affect the final η_*Q*_ result. A schematic comparison of all the experimental systems is presented in Figure 1B–D. We were also aiming to build an optical setup that is simple enough to be easily reproduced in any lab, with as few customized solutions or components as possible.

Most of the studies following the Roper’s concept^34^ exploit a standard 1 × 1 cm cuvette as a sample holder. A significant amount of sample (ca. 2 mL) is required, and the experiment itself is time-consuming (ca. 45 min for heating and 60 min for cooling to reach stable temperature). Simultaneously, it is technically challenging to eliminate long-time drift of light sources, detectors and external temperature. Moreover, it was supposed that the mass input of the cuvette itself, which is a relatively large heat receiver, might disrupt the results. This motivated us to test another setup, originally proposed by Richardson et al.^35^ The cuvette with a colloidal sample was replaced by a droplet of the colloidal sample formed gently with a micropipette tip (Figure 1D). This technically simple approach requires much smaller volumes of colloid, and offers much faster temperature stabilization (<5 min vs >45 min required for the cuvette) during heating and cooling stages. However, because of the high surface-to-volume ratio of the droplet, solvent evaporation cannot be neglected and has proven to affect the actual temperature readout and consequently η_*Q*_. To minimize evaporation, the humidity condition in the measurement chamber was intentionally increased by placing wet blankets inside and sealing the chamber with Parafilm to prevent humidity changes during measurement. A second way to reduce evaporation and also to minimize the impact of specific heat changes as a function of temperature is to intentionally avoid excessive heating, for example, by keeping the temperature rise below 10 degrees during the heating stage and keeping the measurement time as short as possible. Additionally, we paid special attention to keeping the dosing system airtight to avoid droplet regression. The droplet was dispensed with the pipet, and after its formation, a simple mechanical custom-made valve was used to prevent sucking it back. Other details of experimental setup performance are presented in the Materials and Methods and in the Supporting Information (Details of data analysis).

Finally, the calculation of η_*Q*_ requires prior characterization of the optical setup and knowledge of, among others, a mass of the components which stay in direct contact with the heating volume of the nanoparticles. Many reports assume a fixed mass of a whole cuvette for example, but actually Wang et al. proposed a simple and elegant solution for this ambiguous factor.^12^ By using exactly the same optical setup, the optical heating of colloidal nanoparticles was replaced with a Joule resistance wire of known (measured) resistance. It was assumed that electricity is transformed into heat energy with 100% of efficiency, because the setup lacks elements in which energy could be transformed into other forms. Such an approach enabled determination of the product of mass and heat capacities of all system components using eq 8, where the *a* factor is a fitting parameter (in eq 7) achieved for electrical heating calibration, based on heating–cooling kinetic profiles.
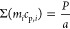
8

From this equation it is possible to evaluate the effective mass of each component, but an assumption that some of them contribute fully in heat exchange is required. For measurements from the top view, it was assumed that colloidal sample mass contributes fully (temperature of sample is homogeneous or close to homogeneous). For the stirring experiments, the full mass of stirrer bar was included (because the stirrer is inside the colloidal sample), and for side-view experiments, the full mass of black tape of known emissivity (directly observed by TGC) was also included. The mass of the heating wire used in the calibration experiments was negligible because its mass of 5 mg and heat capacity equal to 460 J/kg·K (as compared to ∼2g of sample with heat capacity 4180 J/kg·K and ∼6.5 g weight of the cuvete with heat capacity 729 J/kg·K). Therefore, the remaining mass was assumed as an effective mass of cuvette *m*_eff_. This approach also enabled one to minimize the impact of differences in mass of sample due to dosing imperfections (*m* = 2 g ± 0.007 g). Experiments were conducted with different input power (see Calibration Experimental Details in Materials and Methods), so the effective mass of the cuvette was obtained from each experiment independently and then averaged for each configuration. The measurement was carried out for each of the experimental variants separately for at least four different applied current values. The effective mass has been determined independently for each of the measurements to take into account small fluctuations in the mass between these repetitions. Then the obtained effective mass of the cuvette was averaged and these averaged *m*_eff_, individually calculated for a given experimental configuration, were further used in calculations of η_*Q*_ for light induced heating experiments.

### 2.2 Light-to-Heat Conversion Efficiency: The Measurements

To evaluate how various measurement setups, preconditions, and technical or physical assumptions affect the value and accuracy of η_*Q*_ determination, we have decided to study the photothermal properties of gold nanoparticles. AuNPs are one of the most frequently examined light-to-heat converting nanoparticles for hyperthermia treatment because of ease of synthesis, stability in water, biocompatibility,^37^ and high photoinduced heat generation efficiency.^35^ The latter feature originates from localized surface plasmon resonances owing to cooperative oscillations of electrons.^42^ Although the spectroscopic properties of plasmonic nanoparticles strongly depend on their size and shape asymmetry,^43,44^ astonishingly, AuNPs of the same size and shape have demonstrated very different η_*Q*_ values in existing reports,^34,35^ which inevitably means the final η_*Q*_ value depends on the experimental setups, specificities of synthesis made in various laboratories as well as a priori assumptions made during data evaluation. Therefore, the present, and ambiguous, status in photothermal conversion efficiency measurement techniques encouraged us to quantitatively compare the same batch of AuNPs@SiO_2_ sample in different experimental conditions.

**Figure 2:**
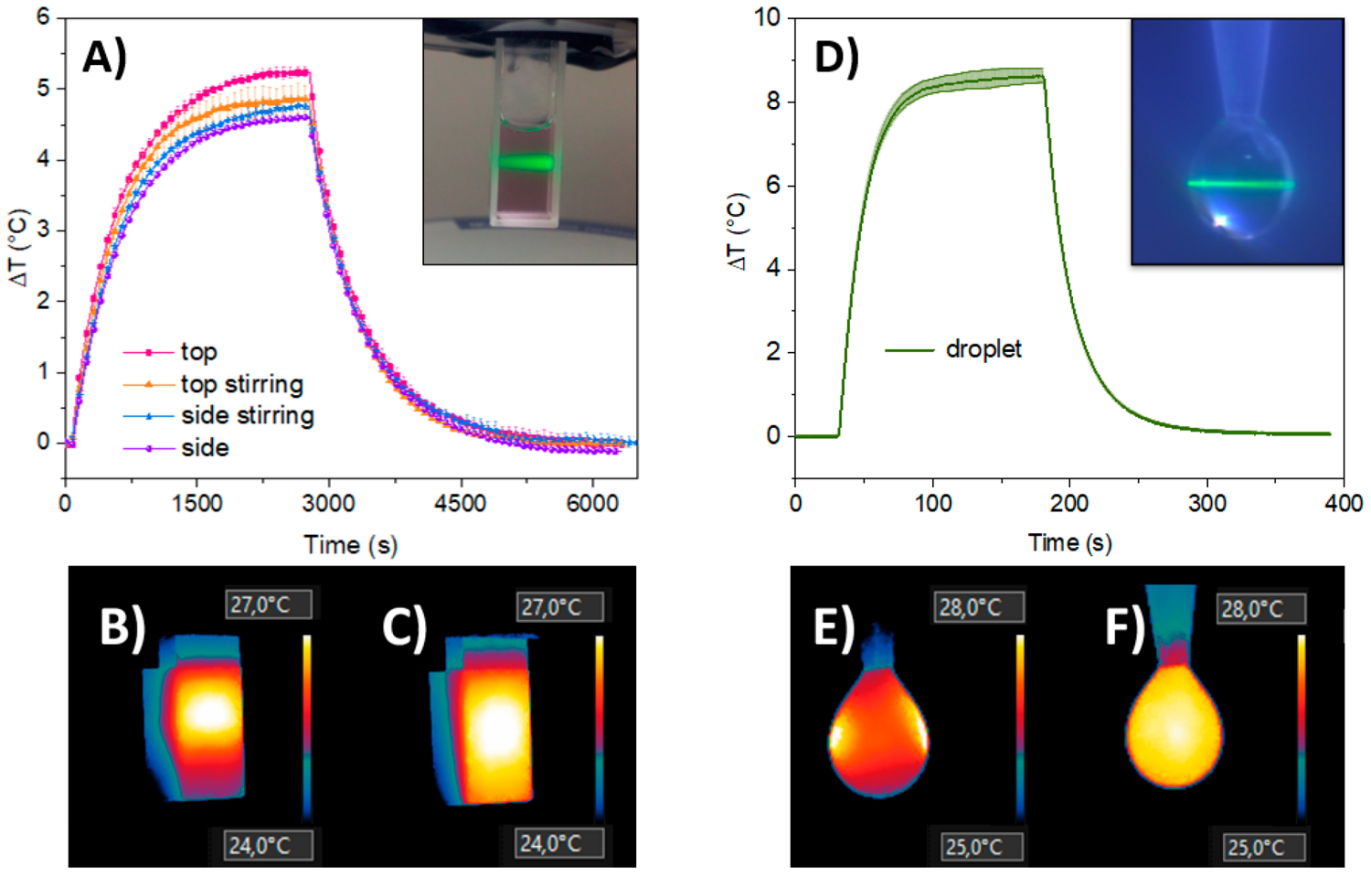
Heating and cooling of Au@SiO_2_ sample in various experimental configurations. (A) Temperature curves in the “standard” setup with TGC in top and side view, with and without mechanical stirring. (B) Corresponding temperature images (obtained from the side view, in a setup without mechanical stirring) at heating (B) and cooling (C) stages. (D) Temperature heating–cooling curves obtained in a setup with droplet and corresponding temperature images of (E) heating and (F) cooling. Description of experimental variants is presented in Figure 1C, experimental parameters are described in SI Table S1.

The η_*Q*_ evaluation (eq 3) requires accurate determination of the (i) absorption coefficient at the photostimulation wavelength, (ii) incident photoexcitation intensity, as well as the (iii) effective mass of the measurement setup, (iv) temperature rise (*T*_max_ – *T*_amb_), and (v) the inverse cooling rate (i.e., cooling time coefficient, τ_c_). While the first two parameters can be easily and precisely measured, the precision of η_*Q*_ determination requires the last three factors be accurately established as well, which is not trivial. For example, in Roper’s experimental setup,^34^ the thermocouple (TC) was located on a surface of a measurement cell directly behind the laser beam, which provides temperature readout close to maximum temperature in a measurement cell. However, the temperature determined in such a way could be underestimated due to the glass cell thickness. In the stationary state, the temperature of nanoparticles and the adjacent media are the same,^45^ thus the temperature of nanoparticles should be measured at a location where the temperature of the colloidal sample is closest to an average temperature. Because of these issues, we have decided to use an alternative approach, by measuring temperature on a surface of the colloidal sample by a thermographic camera (TGC). Although much more costly, the ambiguities related to the positioning of the thermocouple can be neglected. Although the TGC was used before,^46^ the related ambiguity is however associated with the way the *T*_max_ is determined, the highest versus averaged values, or the area of averaging temperature over the heated volume may significantly affect the ultimate η_*Q*_ calculations. This equivocality originates from the presence of temperature gradients (as shown in Figure 2). Our calculations show that considering local maximum temperature and the full mass of the sample in the model proposed by Roper et al.^34^ leads to efficiencies exceeding 100% in some of the evaluated measurement setups (detailed discussion below). In the course of the performed evaluation and optimization, we found out that the most accurate results require averaging the temperature from the whole available sample surface, because the model actually treats the sample as a homogeneous material that naturally exchanges heat with the environment.

**Figure 3:**
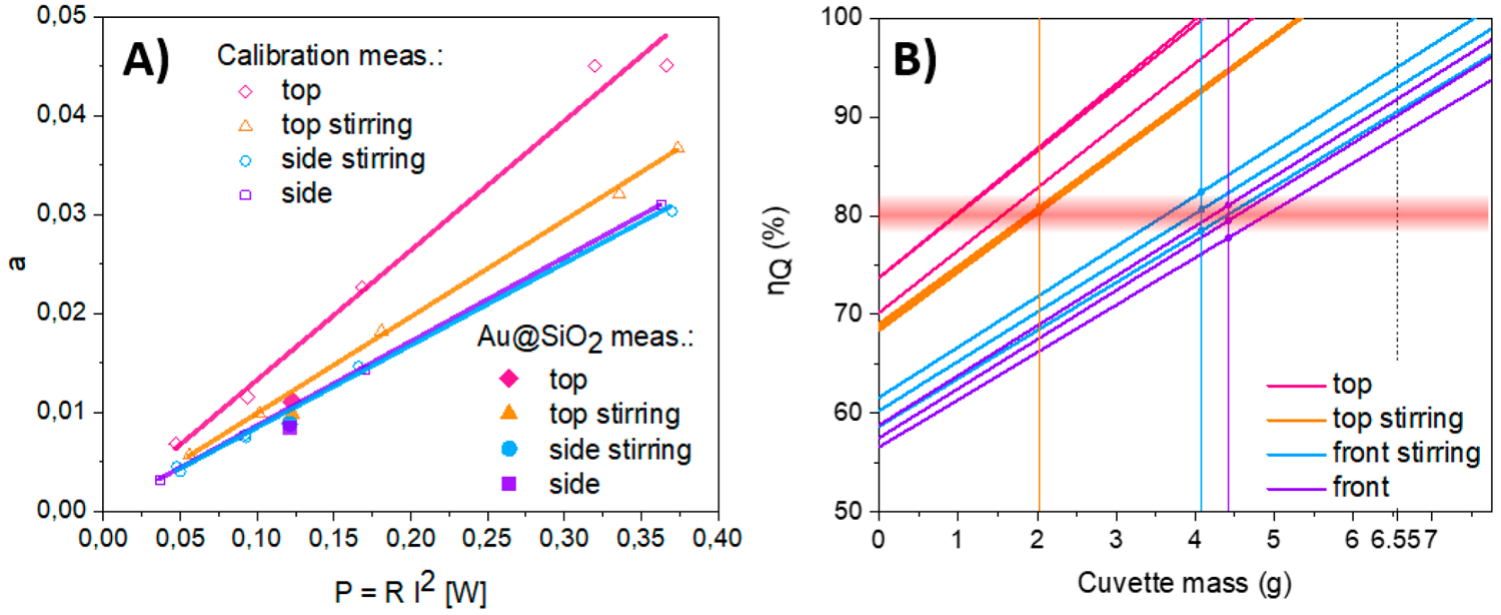
Calibration and experimental light-to-heat conversion data obtained in a spectrometric cuvette (10 mm optical path). (A) Calibration of the *a* factor (to ultimately derive the effective mass) versus power delivered in resistant wire (based on the method presented by Wang et al.^12^) various measurement setups (pure solvent - empty symbols; pink diamond - top view; orange triangle - top view with stirring; cyan circle - side view with stirring; purple rectangle - side view; the corresponding solid symbols represent Au@SiO2 sample). (B) η_*Q*_ in a function of cuvette mass included in calculations: the physical mass of cuvette is 6.55 g; other vertical lines represent calculated effective mass corresponding to particular experimental variants. By including an effective mass correction in data analysis from various setups (top view, pink; top view with stirring, orange; side view with stirring. cyan; side view, purple), coherent η_*Q*_ were found with an average value 80.2% ± 2% for all these presented setups (red diffused horizontal line represents mean value with 2% standard deviation, detailed data of individual experiments are presented in SI Tables S2 and S3). Multiple lines in (B) originate from a few repetitions of the same experiment.

The photostimulation of colloidal nanoparticles by a collimated laser beam will always generate temperature gradients, which are impossible to resolve with a thermocouple having a size comparable to the droplet itself. Moreover, monitoring the temperature with the unsupervised orientation of a thermocouple against the position of the laser beam becomes a serious issue as the beam may accidentally hit and heat the TC directly. Additionally, the same photoexcitation intensity obtained with a Gaussian profile laser beam will generate more heat in the beam center as compared to top-hat profile, thus either conscious data correction must be performed, or temperature gradients must be diminished by mechanical stirring and temperature homogenization.^36,40^ The stirring is additionally expected to speed up the cooling process through an increased dissipation surface, which is actually not included in the most popular Roper’s model. Without stirring, the highest temperature increase is observed in the upper part of a sample due to the convection and heat flow. In general, the fluid convection depends on the system volume and geometry. This phenomenon is difficult to describe numerically in the context of light-to-heat conversion efficiency, but Wang’s setup assumes taking into account the mean temperature of the stirred sample. As a matter of fact, the highest temperature increase is observed on the top of the sample when it is not stirred and when convection occurs (Figure 2A) and that is the reason for differences in calculated parameters *a* and η_*Q*_ when the same mass of system components is assumed for all variants (Figure 3). That makes the efficiency, calculated in conventional way, dependent on stirring.

These observations motivated further modification of the “standard” experimental setup, our aim was to make the measurement independent of convection in a way other than the mechanical stirring of the sample, which is originally not considered in the Roper’s model. For that reason, we have decided to build and evaluate the setup proposed by Richardson et al.,^35^ where the sample is confined to a hanging drop of colloidal nanoparticles, without any container (such as cuvette). However, unlike Richardson, we have measured the temperature increase by TGC with a magnifying germanium lens, instead of using a thermocouple positioned inside the droplet. Our approach increases the costs of the setup, but saves numerous technical and TC position adjustment difficulties, and additionally avoids errors caused by the heat transfer by thermocouple conduction. This last feature is especially appealing, as a small volume of nanoheaters purposely does not heat the sample (i.e., droplet) by more than 10 degrees. Moreover, various ways to derive *T*_max_ can be easily implemented and validated, such as finding the maximum value, or an averaged *T* value over part or the whole *T* image of the droplet. We learned that the most appropriate approach is to average the temperature across the entire observed surface of the droplet. Developing a standardized way to quantify light-to-heat conversion requires quite strict conditions and balancing between numerous factors and issues. The use of the droplet removes all issues related to the impact of sample holder on data acquisition and interpretation, and significantly shortens acquisition time (down to a few minutes per heating and cooling cycle). Another advantage of the droplet system is the fact it can be relatively easily reproduced in other laboratories. The obvious risk is the fact that the droplet evaporates at increased temperatures, thus care must be taken to keep the maximum temperature increase below 10 °C and control humidity within chamber. While the wet towel can be barely considered as a sophisticated scientific tool, in the course of numerous trials and experiments, we came to this simple, yet effective solution and find it most reliable. We have been monitoring the humidity within the sample chamber with a dedicated humidity digital reader, but this knowledge does not allow to correct variation of humidity post factum. In the course of experiments, we have been continuously visualizing the droplet and we noticed the droplet shrinks by ca. 16% after 10 min of the experiment in open humidity chamber, including 2 min of laser beam illumination (resulting at ca. 9 °C temperature increase), while in the same conditions, in increased humidity conditions droplet shrinks by ca. 9%. Intensive shrinkage ends up with shorter laser path within the droplet, increased concentration of colloidal NPs per droplet and other perturbations occurring to the temperature readout by the thermographic camera. And again, knowledge about the decreasing droplet size and the existing models do not allow to correct for this issue post measurement. Concluding, there are many technical challenges related to the optical system design, sample container, calibration, data reproducibility and accuracy, agreement with the model, capability to repeat measurements, etc. that we have verified and discussed in the paper. Our motivations for the research were therefore 4-fold: (1) to directly and quantitively evaluate and compare various setups using the same batch of the sample, (2) to evaluate setups that are sufficiently simple to reproduce in other laboratories without sophisticated, costly and complex optical setups, (3) to evaluate light-to-heat conversion nanoparticles that are dedicated for biomedical hyperthermia, (4) to enable fast and easily repeatable measurements with small volumes of NPs water colloids. Targeting specifically hyperthermia application means that temperature increase of 10 degrees from the initial temperature should be sufficiently broad operation temperature range. This apparent restriction has one another important advantage, namely, low thermal stability at high temperatures found in many of existing photothermal agents can be disregarded from analysis. In consequence of the assumptions we made, many other problems can be avoided, for example η_*Q*_ should be independent from the operating temperature range (the verification experiment of the temperature impact on η_*Q*_ is shown in SI Figures S1 and S2). Moreover, the potential specific rates of thermal decomposition of various PTT agents can be excluded from the analysis, which simplifies the interpretation and quantitative comparisons as well as disable the misestimation of the light-to-heat conversion efficiency for such thermally unstable photothermal agents. Hence, the estimations of the η_*Q*_ upon lower excitation power in standardized conditions, as presented in this manuscript, should enable reliable quantitative comparison between various nanoheaters. However, based on the calculated η_*Q*_ for given material and using the eq 6, one may easily extrapolate and predict the temperature rise at increased concentration of nanoheaters, or at increased pumping intensity, when higher temperatures must be obtained as during PTT. We therefore believe that the proposed method is a very simple and effective way of circumventing the above-discussed issues.

Besides the issues discussed above, there is still no agreement in the literature about which component masses of the measurement setup should be included in eq 3. According to the original Roper’s model,^34^ all elements should be incorporated (i.e., the whole colloidal solution of NPs, the cuvette and even the thermocouple itself) and this interpretation was further frequently reproduced in other works.^47,48^ Some other approaches, like in the droplet model by Richardson,^35^ included only the mass of the colloidal solution and excluded the cuvette mass from calculations.^44,49−51^ Marin et al. evaluated light-to-heat conversion from a theoretical perspective, and concluded that excluding the cuvette mass from calculations resulted in experimental η_*Q*_ being closer to the calculated theoretical value.^40^ The importance of this factor is evident, as the results from the simulation presented in this work differ by almost 30% for the same sample with different setup characterizing data. Alternatively, Lindley and Zhang^10^ suggested that only the part of the cuvette in direct contact with the colloidal nanoheaters solution should be included in the evaluation to avoid overestimation of the η_*Q*_ value. Another alternative way of solving that issue was proposed by Wang et al.,^12^ who proposed to experimentally establish the “effective” mass of a cuvette that participates in heat exchange and should actually be included in the calculations. The most important differences in existing measurement assumptions related to the various models are presented in Table 1 and SI Table S5. Additionally, details about the Chen’s model are presented in the SI (Chen’s model). All these above-described models are frequently referred to in publications that aim to present new nanomaterials or make a comparison of existing ones (e.g., Roper,^15,47−49,51,52^ Chen,^10,16^ and Wang^53^). Replacing the colloidal nanoheaters by an electrically driven resistance wire phantom, Wang discovered that only, ca. 20% of the bona fide mass of the cuvette actually had to be included in the data analysis. This value may vary depending on a particular configuration, but (i) because the electrically driven wire phantom has a known resistance and efficiency, (ii) it is power supplied with easily measurable current, and (iii) because it is studied with exactly the same sample holders and detection setup configuration as in the light-to-heat conversion measurements, the calibration procedure is simple to implement and enables one to account for the effective mass of the sample holder. In our measurements, we applied a similar calibration procedure using the same experimental conditions as those further used to quantify light-to-heat conversion efficiency with nanoparticle heaters (Figure 3A), which enabled us to compare the efficiencies obtained by different methods for the same sample (Figure 3B). As expected, the calculated “effective mass” showed some degree of variability depending on the actual measurement configuration (Figure 3A). For “top” measurement variants, the heating rate is dependent mostly on convection or mechanical stirring of the sample, so the impact of the cuvette is lower than in “side” variants, in which the heat conduction through the wall of a quartz cuvette plays an important role. The comprehensive comparison of the above-described methods of light-to-heat efficiency, performed for the same batch of AuNP@SiO_2_ is presented in Figure 3B. The obtained efficiencies are compared in Table 2 with other literature reports on ca. 20 nm diameter spherical Au nanoparticles. The comparison of efficiency of other plasmonic and nonplasmonic nanomaterials is presented in research works.^9,54^ Detailed (not averaged) experimental data are presented in SI Tables S2–S4. The results obtained with the Roper’s model vary considerably. The η_*Q*_ for the “top” configuration is the highest among all the obtained with this model. The most probable reason for η_*Q*_ to exceed 100% is generation of significant temperature gradients and the fact this is not the entire volume of the sample that gets heated homogeneously, but the heating is induced and monitored only in its top part. This result is closest to Richardson’s result, however Au@SiO_2_ NPs are expected to have lower light-to-heat conversion efficiency than Au NPs because the coating leads to higher scattering due to the increase in size. It is worth noting that results obtained with a modified Wang’s model lead to the same η_*Q*_ value, independently from the measurement conditions. Only in the “top” variant, the η_*Q*_ is slightly lower, but this is probably caused by difficulties in reproducing the laser beam by a heating wire: cables connecting the heating wire with the current leads were attached from above, and thus can disturb the precise determination of the effective mass in this variant. This case indicates how important it is to correctly determine the effective mass in an irregular geometry system. On the other hand, the effective mass of the pipet tip, which served to create the droplet was found negligible, as expected.

**Table 1:** Features, Advantages, and Drawbacks of Existing Experimental Setups for η_*Q*_ Measurements^a^

reference	sample holder	stirring	external	T detector	issues/challenges	advantages
Roper et al^34^	specialized glass cell	no	yes	TC on the glass wall behind the laser beam	homemade small volume experimental cell; vacuum chamber	measurement in the vacuum eliminated heat exchange
Richardson et al^35^	bare droplet formed with syringe	no	no	TC above the laser beam	evaporation of the droplet; droplet retraction	small amount of sample required; short measurement time
Chen et al^36^	cuvette	yes	yes	TC inside the cuvette	large amount of sample required	homogeneous temperature distribution
Wang et al^12^	cuvette	yes	effective mass	TC inside the cuvette	the need of calibration of the system	homogeneous temperature distributioncalibration assures quantitative evaluation
this work (based on Richardson setup)	bare droplet formed with syringe	no	no	TGC		small amount of sample required; short measurement time; inhibited droplet evaporation
this work (based on Wang setup)	cuvette	yes/no	“effective mass”	TGC in “side” or “top” configuration	the need of calibration of the system	homogeneous temperature distribution; calibration assures quantitative evaluation

a TC, thermocouple; TGC, thermographic camera; the “external” column indicates whether the model includes the mass of external system components such as cuvette, stirring bar, etc.

**Table 2:** Comparison of η_*Q*_ Obtained for Various Diameter Spherical Au Nanoparticles^a^

material	NP size (nm)	λ_EXC_ (nm)	m_EFF_	temperature detector	experimental setup comments	model	η_*Q*_ (%)
Au NPs^34^	20	514	whole glass cell	TC outside cell	small sample cell in a vacuum chamber	R	3.4–9.9
Au NPs^35^	20	532	droplet	TC inside droplet	sample is a droplet	R	100
Au NPs^50^	15	532	solvent only	TC inside cuvette	MS, open cuvette	R/C	78.4
Au nanospheres–theoretical abs/ext value^50^			–		–	–	99.6
Au@SiO_2_ nanospheres [this work]	13 ± 2 (Au), ∼ 140 (Au@SiO_2_)	532	solvent only	TGC; through glass	MS on	R	57.5
					MS off		50.8
			solvent only	TGC; sample surface temperature	MS on	R	63.1
					MS off		67.5
			cuvette included	TGC: through glass	MS on	R	90.5
					MS off		80.8
			cuvette included	TGC sample surface temperature	MS on	R	98.4
					MS off		106.7
			“effective mass”	TGC through glass	MS on	W	**80.5**
				TGC sample surface		W	**80.6**
			droplet	TGC sample surface	sample is a droplet	R	66.8
						W	**81.1**

a The following abbreviations were used for the models: R, Roper’s model; R/C, Roper’s model with Chen’s modification (stirring); W, Wang’s model; TC, thermocouple; TGC, thermographic camera; MS, magnetic stirring; λ_EXC_, irradiation wavelength; *m*_EFF_, part of mass of a cuvette.

## 3 Conclusion

Light-to-heat conversion efficiency is one of the most important figures-of-merit in the studies of materials dedicated to the photothermal therapy of cancer. The η_*Q*_ should enable one to quantitatively and reliably compare, and further optimize, various nanoheaters between various laboratories. Unfortunately, large discrepancies in η_*Q*_ are commonly found even for very similar nanoheaters, making this quantity unreliable for its purpose. Through careful evaluation of theoretical models, numerous assumptions and experimental setups made for the same batch of AuNPs@SiO_2_ nanoheaters, we have critically evaluated existing systems and models, and thus contributed to the understanding and standardization of η_*Q*_ determination. More specifically, despite the fact that η_*Q*_ is nominally a material constant, its determined value was found to depend on numerous measurement conditions and assumptions, such as the mass of the system and its geometry, the presence of colloidal sample stirring, and the specifics of how and where the temperature was measured. Before a ranking of different HTNPs can be reliably made, a unified approach to η_*Q*_ is a prerequisite.

Our measurements confirm that (simple to implement) effective mass correction enables to obtain coherent and directly comparable η_*Q*_ values even though significantly different measurement setups were applied. Alternatively, we have also critically evaluated and presented an improved measurement system exploiting the droplet based concept, which not only eliminated the need for effective mass correction, but most of all reduced the volume of the sample ca. 100-fold and reduced recording time up to 10-fold as compared to other conventional measurement systems. Additional modifications, aiming to reduce sample evaporation during measurements were proposed to further minimize artifacts and improve the reliability of the obtained efficiency values.

## 4 Materials and Methods

### Au@SiO_2_ Preparation

Gold(III) chloride hydrate (99.995%), tetraethyl orthosilicate-TEOS (99%), ammonia solution (28–30%), were purchased from Sigma-Aldrich. Ethanol (96%) was purchased from Avantor and sodium citrate was purchased from MERCK. All chemical reagents were used without further purification.

**Figure 4:**
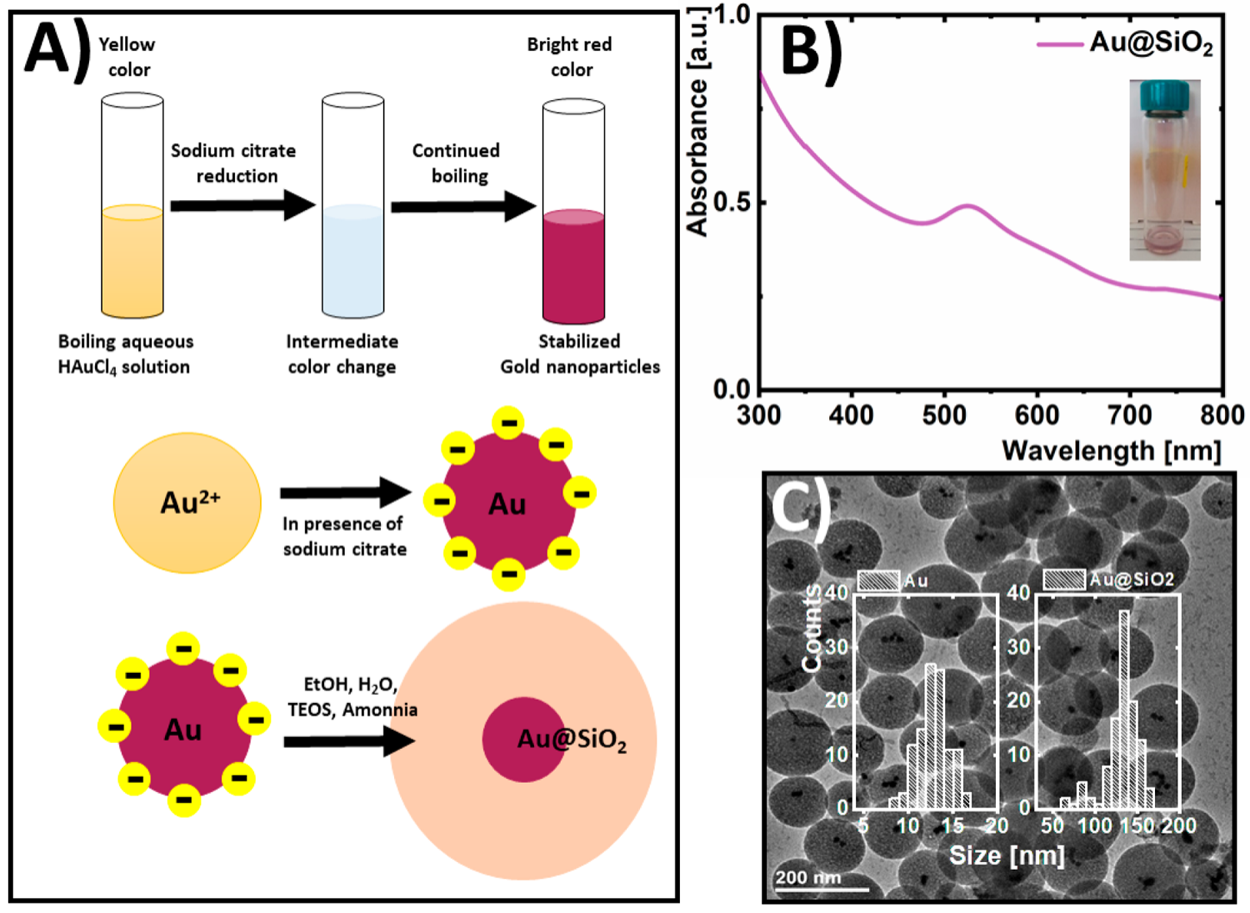
(A) Scheme of two-step synthesis of Au and Au@SiO_2_ nanoparticles, (B) absorption spectra of the colloidal solution of Au@SiO_2_ nanoparticles in water, (C) TEM images with size distribution of pristine and SiO_2_ covered AuNPs.

Au NPs were prepared by reduction the gold salt with sodium citrate.^55,56^ The freshly prepared water solution of sodium citrate was added to 9.95 mL of water solution of HAuCl_4_ in constant temperature (80 °C) under vigorous stirring. The color of the mixture turned wine red after few minutes which indicates the production of Au nanoparticles. The protocol of synthesis is illustrated in Figure 4.

Then, Au NPs were coated with silica. The as-prepared Au nanoparticles were mixed with a proper amount of ethanol (12 mL), distilled water (500 μL), TEOS (20 μL), and ammonia (500 μL). The solution was maintained at room temperature for 24 h under vigorous stirring. Next, the mixture was centrifuged (10 000 rpm, 10 min) and the obtained pellet was purified by ethanol and dispersed in distilled water.

The photostability was checked independently (SI Figure S3). Four cycles of heating and cooling were performed on the same droplet of AuNPs from the same batch as previously. Sample was diluted before experiment to decrease maximum temperature and reduce evaporation. Results have shown that maximum temperature was not decreasing in the next cycles.

### Experimental Details

The first experimental variant is based on the Roper’s setup.^34^ It consists of the activation light source–laser diode (532 nm, 1 W, Changchun New Industries Optoelectronics Technology Co. Ltd.), optical setup for photon beam collimation, fluorescence cuvette containing 2 mL solution of nanoparticles, thermographic camera (TGC) (FLIR T540, accuracy ±0.5 °C with a reference, thermal sensitivity <40 mK, 24° @ 30 °C) and thermo-higrometer (ETI Ltd., type 6500). The cuvette was inserted into the holder which remained in minimal contact with the upper part of the cuvette to reduce heat transfer to other elements of the system. The cuvette entrance wall was set at the angle of 7° to the laser beam to avoid multiple reflections, and the exit wall was set at the angle of approximately 15° to the TGC to eliminate camera own reflections from the perpendicular wall of the cuvette. This wall of the cuvette was covered with piece of black tape of known emissivity (*E* = 0.96, *m* = 0.15 g, 3M, Poland) for accurate measurement with a TGC. The system was isolated from external light and heat by a 5 cm thick styrofoam cage. Measurements were conducted in an air-conditioned room at 23 °C and constant humidity conditions during all experiments. The laser power of 200 mW was set and mean power density was 1.6 W/cm^2^. The laser beam (spot size 4 mm) was hitting the center of the entrance wall under 7° angle, to avoid multiple reflections in quartz cuvette.

Because significant heating can be expected for highly concentrated samples due to plasmonic effects, we measured our samples in diluted form (to keep heating of no more than 10 °C above RT) and indeed in the course of the experiments we did not observe any concentration dependent effects. In a typical measurement “side-view” procedure (Figure 1B), the sample was dispersed on the ultrasonic scrubber and inserted (2 mL, 2 cm height) into the cuvette, which was fixed into stable position by cuvette holder. The focus of the TGC was set to the side surface of the cuvette (90° angle to the laser beam axis) and was kept the same for all the measurements. Then the setup was left for approximately 20 min to reach stable temperature distribution in the field of view of the TGC. The laser diode was turned on at least 2 min before each experiment, but the beam was blocked by a mechanical shutter to prevent sample illumination before the actual experiment started. Recording temperature images by TGC and recording laser power with power meter (photodiode S120C head and PM100USB power meter, Thorlabs) started simultaneously. After 70 s the laser beam shutter was unlocked and heating curves were registered. Then, after 45 min laser was turned off and the cooling curve was registered for the next 60 min.

Data from the TGC was analyzed in FLIR Tools software. Temperature of sample was averaged from the whole cuvette surface area staying in direct contact with the colloid. The η_*Q*_ calculations derived from the cooling curve were based on equations presented by Roper et al.,^34^ while the ones derived from the heating curve, were using equations presented by Wang et al.^12^ Other details of data analysis and well as error analysis are presented in SI (Details of data analysis). Because in the described setup significant gradients of temperature were noted in the initial phase of heating–cooling kinetic profiles, we verified the impact of gentle magnetic stirring on the output temperature profiles quality. For that purpose, the magnetic base was placed below the cuvette bottom (without direct contact), and a small magnet bar (3 × 1 × 1 mm) was dropped into the cuvette. The stirrer was turned on 1 h before the experiment started to obtain a stable temperature gradient in the sample environment.

Next the experimental setup was modified to provide direct colloidal surface measurements (“top view” variant, Figure 1C). TGC was positioned above the experimental setup with a 9° angle between objective axis and colloid surface. Black tape of known emissivity was removed from the cuvette because it was not required in this setup configuration (water emissivity is known, *E* = 0.90). In this case, the temperature recorded by the camera was acquired through the round neck of the cuvette, thus the temperature was averaged from ellipsoid part of the TVC image (whole available sample surface). Similarly to the “side view” experimental setup, the influence of magnetic stirring was verified.

Experiment with “droplet” configuration required similar optical setup, but it requires also independent sample dosing system (SI Figure S4). Also measurement procedure was more complicated in this case (see SI Droplet setup-detailed experimental procedure and issues).

### Calibration Experimental Details

Instead of a laser beam, 0.3 mm kanthal resistance wire of known (measured) resistance was used. The current was supplied to the heating wire using silver-plated copper wire. The wire was carefully fixed at position, where photostimulating laser beam was heating the sample to keep the position of the TGC and all other components in the given configuration exactly the same as during photostimulation experiments. The heating element was welded to silver-plated copper wire to obtain a stable electrical connection. A current of 0.25–1.02 A was set on the regulated DC power supply (MCP M10-QS3020, Poland) and the voltage on the heating wire was recorded with a multimeter (attached to the wire slightly above the height of the cuvette). Based on this data, resistance (around 380 ohms) and supplied power (P) were determined.

## Supporting Information Available

Standardization of light to heat conversion efficiency; The CAD drawing of the chamber (suitable for 3D printing) for the droplet setup is available from us upon request (PDF)
