# Supplementary material for: Standardization of Methodology of Light-to-Heat Conversion Efficiency Determination for Colloidal Nanoheaters
Source: ACS Appl Mater Interfaces. 2021 Sep 9;13(37):44556–67. doi: 10.1021/acsami.1c12409 (PMC8461604; doi:10.1021/acsami.1c12409)
Supplement: Supplementary file 1 — am1c12409_si_001.pdf [file am1c12409_si_001.pdf]

Supporting Information

Standardization of Methodology of Light-to-Heat Conversion Efficiency Determination for Colloidal Nanoheaters

Agnieszka Paściak[1], Aleksandra Pilch-Wróbel[1], Łukasz Marciniak[1], Peter James Schuck[2],

Artur Bednarkiewicz[1]*

[1] Institute of Low Temperature and Structure Research, Polish Academy of Sciences, Okólna 2, 50-422 Wroclaw, Poland

[2] Department of Mechanical Engineering, Columbia University, New York, NY, United States

* Author to whom correspondence should be addressed: a.bednarkiewicz@intibs.pl

Details of data analysis

Droplet size was determined from thermographic camera data in TGC camera FLIR Tools software: the number of pixels forming a drop was determined and based on the scale (i.e. plastic tip end diameter) the real droplet volume was calculated. Temperature was averaged from the whole available droplet surface excluding edges (pixels in temperature range between temperature of droplet and temperature of background). Due to the fact that the thermographic camera accuracy is 2 °C, we made efforts to improve this value by concurrently taking the reference measurement of a background, which remained at a constant temperature and we have subtracted the noisy background signal. As we have checked, this operation did not introduce any additional measurement errors, and allowed to eliminate artifacts, such as imperfections of the thermographic camera. In the case of a droplet, wet tissues placed nearby the droplet were chosen as background measurement area, to include the temperature difference between droplet and environment caused by water evaporation. In case of measurements in cuvette, the background signal originated from black tape above the

cuvette (black tape was also placed on the cuvette to minimize reflections from quartz glass cuvette). Data from FLIR Tools and Thorlabs optical power meters was exported, and then analyzed in Origin 2019 software.

Droplet setup – detailed experimental procedure and issues

The optical power calibration measurement was carried out to determine the ratio between reference and measurement optical power. The dosing system was sealed and refilled with water to avoid uncontrollable droplet regression or ejection. Thermographic camera was set perpendicular to the droplet dispensing system. Humidity chamber was also optimized – a vessel with water has been placed below the droplet and tissues soaked with water were placed nearby. Water reference measurements were conducted before and after sample measurements. Before experiment, sample was dispersed on the ultrasonic scrubber. After dropping, the position of droplet was adjusted and the chamber was isolated with Parafilm. Beam shutter was set in OFF position and the whole setup was covered by Styrofoam and black fabric. Power meters were zero adjusted and the setup was left alone until the temperature difference between the droplet and surroundings was less than 0.2 degree, which indicated that the humidity inside the chamber was stabilized. Then the laser was turned on and after 2 minutes the measurement by thermographic camera and power meters started. Initial 30 s were "dark" measurement, then the shutter was removed and the sample was heated by laser irradiation. After 3 minutes, when the temperature of the droplet became stable, the laser was turned off, and sample was cooling down. After 7 minutes when temperature turned back into initial state, the measurements were stopped. All further data analysis was performed off-line.

We have made effort to make the system possibly reliable. We have resolved many experimental issues:

1. We found out that various nanomaterials might have different contact angles, so the maximum droplet size may differs. It also may depend on surface charge of the pipette tip. The system has to include droplet position regulation (we have built simplified system which allows for that, but we recommend to use XYZ stage).

2. Droplet acts like a lens, so its position has to be fixed in the way that enables light transmission measurements throughout the exact Z-axis of the setup.

3. We found out, that our pipette itself is not tight enough to preserve droplet from retraction. To solve that, firstly we have tried to use solenoid valve, but it generated significant amount of heat which disrupted measurements. From that reason we have decided to use a mechanical valve (home-made one, commercially available ones should also work). We found out that the system is tight when all connections are filled with water.

4. Droplet is relatively small object (no more than 3 mm in diameter), so to register distinct picture of it, thermographic camera (FLIR T540) with a macro IR lens should be located close enough. Because the power supply electronics of the camera generates heat which through specular reflection from flat gall surfaces may affect the measurements, we have decided to use magnifying germanium lens (Ø=1", F=50 mm, Thorlabs) with transmission matching the transmission of the TGC camera lens.

5. We have discovered that humidity plays significant role in temperature measurements, especially when the amount of sample is small: we have built a humidity chamber to minimize the evaporation.

Power dependence of Au@SiO_2 nanoparticles light-to-heat conversion efficiency

The measurement of light-to-heat conversion efficiency as a function of laser power was carried out in the droplet system using originally synthesized NPs about 1.5 years after the

sample synthesis. Some changes were observed in samples properties (Figure S1) such as: smoothing of the peak at approx. 600 nm, reduced scattering (these changes are probably due to sedimentation of synthesis residues or aggregates on the bottom of the sample) and a relatively higher peak height corresponding to the maximum of plasmon resonance (which could be caused by sample evaporation). During experiments (Figure S2) the temperature increased by no more than 9 °C above room temperature for pump laser power in the 9 to 118 mW range. The obtained results display very similar values, which stay within measurement accuracy and neither a rising nor falling trend could be observed. High standard deviations for several points are mainly caused by the dispersion of droplet sizes, which was monitored during the experiments with the calibrated thermographic camera.

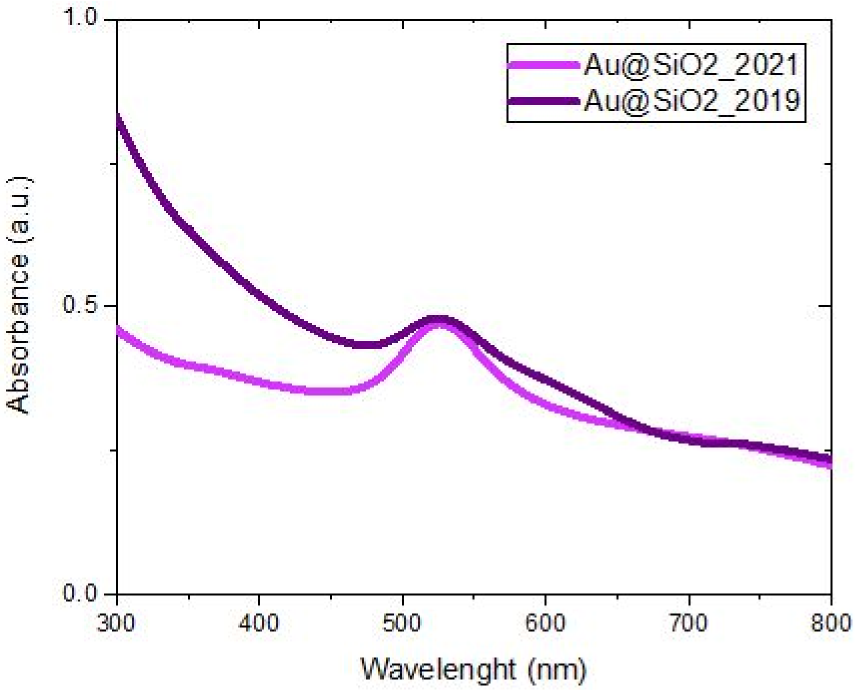

Figure S1. Absorption spectra of the sample used for power dependent η_Q experiment: original and freshly measured (dark purple) and 1.5 years old (light pink) samples.

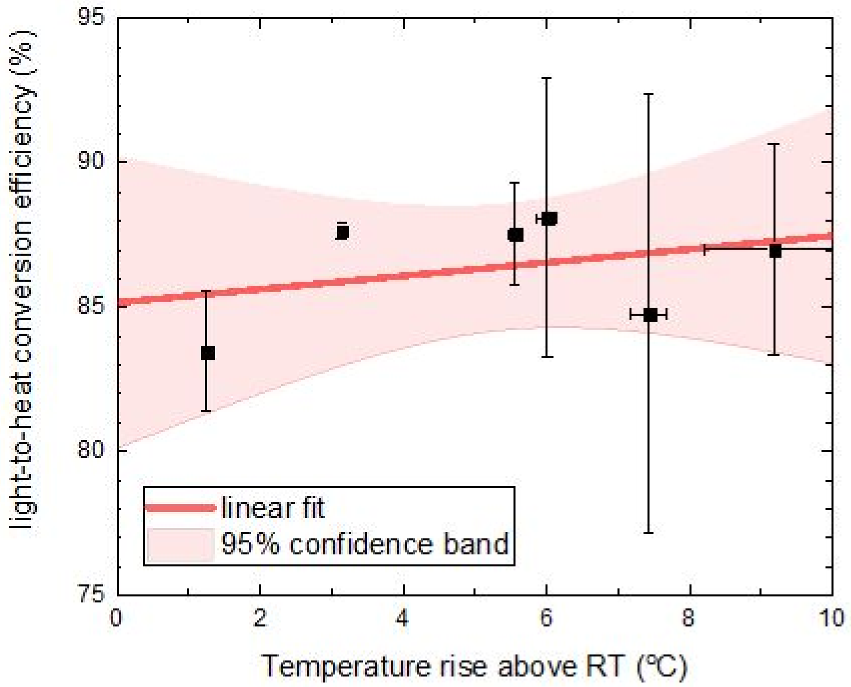

Figure S2. Dependence of the light-to-heat conversion efficiency on self-generated temperature for the Au@SiO_2 sample. The error bars show standard deviation calculated from at least 3 measurements.

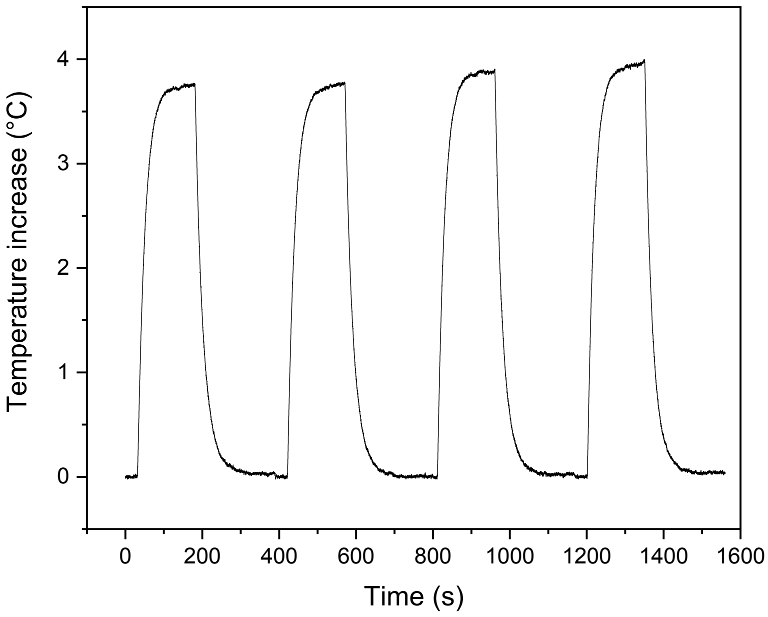

Figure S3. Heating and cooling curves of diluted $Au@SiO_2$ sample: laser power: 116.9 mW, sample absorbance: 0.054, sample mass: 10.8 μg

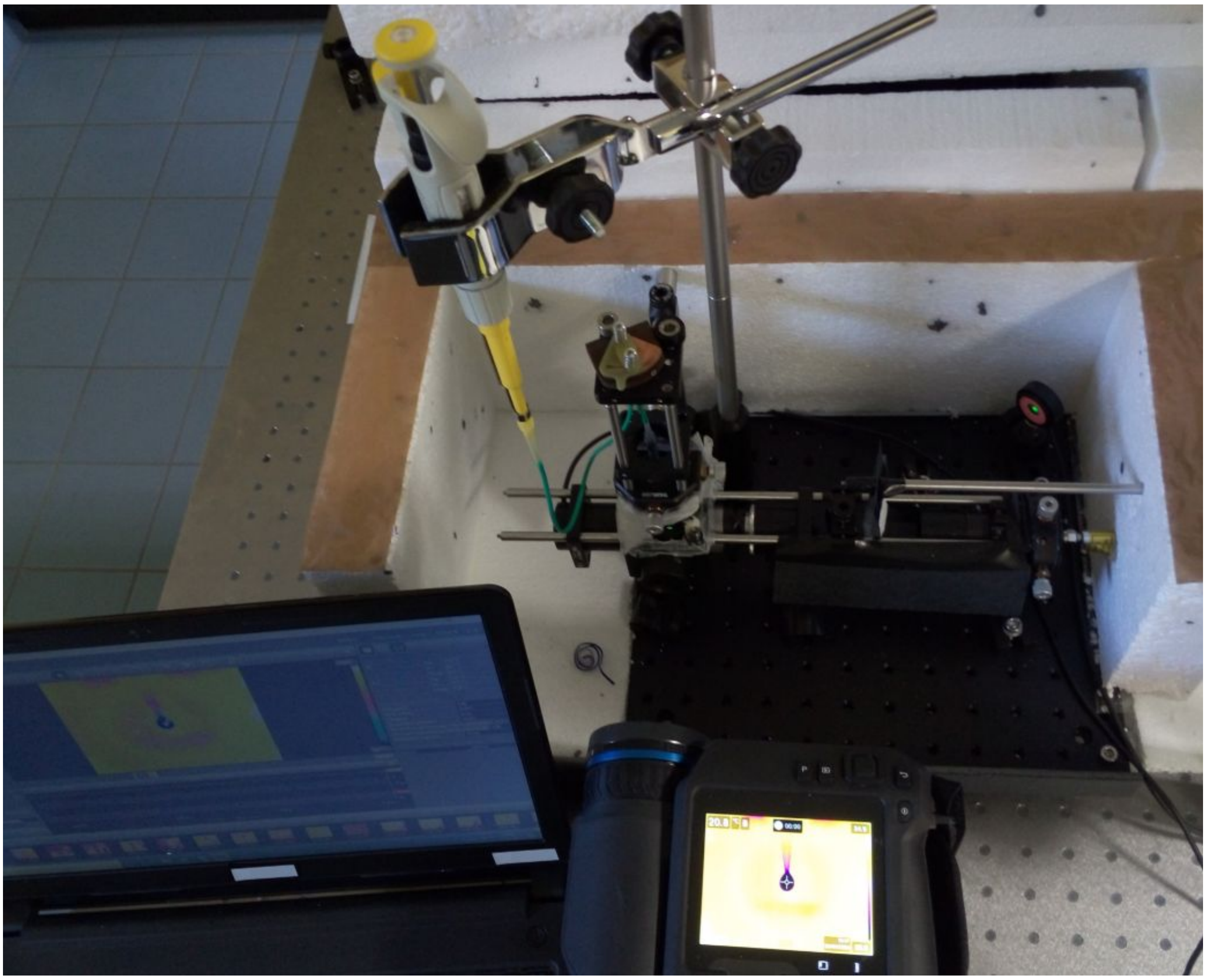

Figure S4. Droplet experimental setup photography

Table S1. Experimental parameters

Experiments in cuvette:

Main parameters	
Type of excitation	Continuous wave
Laser power (mW)	~200
Power density ($\frac{W}{cm^2}$)	~1.6
Laser contact surface (mm^2)	~12.6 (FWHM=4mm)
Q_0 ($\frac{J}{s}$)	0
C_{sample} ($\frac{J}{kg*K}$)	4190

Thermovision parameters	
Emissivity (-)	0.90 / 0.96
Camera angle (°)	7
Distance (m)	0.2
Humidity (%) (laboratory)	~50
Air temperature (°C)	23
Reflected temperature (°C)	23

Droplet experiment:

Main parameters	
Type of excitation	Continuous wave
Laser power (mW)	~115
Density power ($\frac{W}{cm^2}$)	235
Laser contact surface (mm^2)	0.049
Q_0 ($\frac{J}{s}$)	0
C_{sample} ($\frac{J}{kg*K}$)	4190

Thermovision parameters	
Emissivity (-)	0.9
Camera angle (°)	0
Distance (m)	0.1
Humidity (%) (chamber)	~80
Air temperature (°C)	23
Reflected temperature (°C)	23

Table S2 Detailed data of experiments performed in cuvette, the ± values are standard deviation of the obtained results.

Exp.	dT	Abs [-]	I [mW]	Tau decay [s]	Tau rise [s]	Sample mass [g]	a (Wang)	Efficiency – Roper (m=mass of sample + stirrer bar if used)		Effective mass of the cuvette [g]	Efficiency – Wang (effective mass)	
**Side 1**	4.62	0.417	198.9	613.5	579.8	2.02	0.00808	51.8	50.8 ± 1.6	4.42 ± 0.25	77.8	79.4 ± 1.7
**Side 2**	4.65	0.417	197.6	617.3	558.6	2.00	0.00842	51.7			81.0	
**Side 3**	4.56	0.417	197.1	632.9	551.9	1.98	0.00830	49.0			79.5	
**Top 1**	5.87	0.421	199.7	591.7	545.7	2.01	0.01088	67.3	67.6 ± 0.4	-1.16 ± 0.77	66.3	65.1 ± 2.0
**Top 2**	5.98	0.421	199.9	584.8	544.9	1.97	0.01111	67.9			66.2	
**Top 3**	6.04	0.421	198.6	588.2	568.0	1.94	0.01067	67.5			62.9	
**Side Stirring 1**	4.83	0.402	201.0	574.7	598.6	2.01	0.00808	59.8	57.5 ± 2.3	4.08 ± 1.05	78.5	80.5 ± 2.0
**Side Stirring 2**	4.67	0.402	200.4	581.4	552.7	2.01	0.00846	57.3			82.4	
**Side Stirring 3**	4.70	0.402	203.9	595.2	561.8	2.01	0.00842	55.3			80.6	
**Top Stirring 1**	5.08	0.421	201.2	546.4	512.6	2.01	0.00999	64.2	63.1 ± 1.2	2.02 ± 0.36	80.9	80.6 ± 0.3
**Top Stirring 2**	4.96	0.421	197.2	549.5	506.3	1.99	0.00985	63.1			80.6	
**Top Stirring 3**	4.99	0.421	197.7	552.5	505.7	1.96	0.00996	62.0			80.3	

Table S3. Detailed results of Wang's effective mass calibration experiment. For all experiments the same cuvette (6.5g) has been used. Values of specific heat capacity included in calculations were: c (H_2O)= 4180 $\frac{J}{kg*K}$; c (quartz glass) = 729 $\frac{J}{kg*K}$; c (PCV - tape) = 1172 $\frac{J}{kg*K}$. Resistance was evaluated as a product of voltage and current minus input wire resistance, ± values are standard deviation of obtained results.

Exp.	I [A]	R [ohm]	$P = RI^2$ [W]	Sample mass [g]	Tape mass [g]	Stirrer bar mass [g]	a (Wang)	Effective mass of the cuvette [g]	Effective mass of the cuvette [g]
**Side 1**	0.32	0.35863	0.03672	2.020	0.171	0	0.00312	4.26	4.42 ± 0.25
**Side 2**	0.69	0.35749	0.17020	2.001	0.171	0	0.01437	4.47	
**Side 3**	1.00	0.36300	0.36300	2.018	0.171	0	0.03098	4.20	
**Side 4**	0.50	0.36900	0.09225	2.013	0.171	0	0.00763	4.74	
**Side Stirring 1**	1.01	0.36280	0.37009	2.016	0.171	0.197	0.03033	4.56	4.08 ± 1.05
**Side Stirring 2**	0.50	0.37100	0.09275	2.009	0	0.197	0.00747	5.17	
**Side Stirring 3**	0.67	0.36987	0.16603	2.008	0.171	0.197	0.01468	3.38	
**Side Stirring 4**	0.50	0.36900	0.09225	2.014	0.171	0.197	0.00775	4.16	
**Side Stirring 5**	0.36	0.38744	0.05021	2.025	0.168	0.197	0.00403	4.85	
**Side Stirring 6**	0.35	0.39014	0.04779	2.012	0.168	0.197	0.00452	2.35	
**Top Stirring 1**	0.93	0.38816	0.33572	2.012	0	0.197	0.03204	2.49	2.02 ± 0.36
**Top Stirring 2**	0.99	0.38138	0.37379	2.008	0	0.197	0.03672	2.11	
**Top Stirring 3**	0.68	0.39153	0.18104	2.013	0	0.197	0.01823	1.74	
**Top Stirring 4**	0.51	0.39006	0.10145	2.019	0	0.197	0.00990	2.14	
**Top Stirring 5**	0.38	0.38774	0.05599	2.013	0	0.197	0.00569	1.61	
**Top 1**	0.91	0.38586	0.31953	2.006	0	0	0.04502	-1.79	-1.16 ± 0.77
**Top 2**	0.50	0.37500	0.09375	2.008	0	0	0.01156	-0.42	
**Top 3**	0.66	0.38694	0.16855	1.998	0	0	0.02266	-1.28	
**Top 4**	0.35	0.38586	0.04727	2.008	0	0	0.00679	-1.99	
**Top 5**	0.98	0.38178	0.36666	1.998	0	0	0.04508	-0.33	

Table S4. Detailed data of experiment performed in droplet setup; the ± values are standard deviation of the obtained results.

Exp.	dT	Abs [-]	I [mW]	Tau decay [s]	Tau rise [s]	Sample mass [µg]	a (Wang)	Efficiency – Wang [%]	Efficiency – Roper [%]
Drop 1	8.41	0.167	115.6	21.2	17.0	13.7	0.493	85.9	69.3
Drop 2	8.86	0.180	117.5	21.1	17.4	14.1	0.509	83.1	68.5
Drop 3	8.62	0.175	117.2	21.5	17.5	14.4	0.492	80.4	65.6
Drop 4	8.44	0.182	113.3	21.6	18.4	14.4	0.456	76.7	65.7
Drop 5	8.66	0.182	118.0	21.8	17.8	14.1	0.484	79.5	65.1
mean	8.60 ±0.19	0.177 ±0.007	116.3 ±2.0	21.4 ±0.3	17.6 ±0.6	14.1 ±0.3	0.487 ±0.020	81.1 ±3.6	66.8 ±2.0

Elaboration of experimental errors on calculations of light to heat conversion efficiency in the droplet configuration

Equation (6), which is used to determine light-to-heat conversion efficiency, was supplemented with Q_0 - heat flow induced by absorption of light by solvent:

$$\eta = \frac{a\sum mc_p - Q_0}{I\left(1-10^{-A_\lambda}\right)} \tag{S1}$$

The measurement error was estimated using the total differential method:

$$\Delta\eta = \left|\frac{d\eta}{da}\right|\Delta a + \left|\frac{d\eta}{d\sum mc_p}\right|\Delta\sum mc_p + \left|\frac{d\eta}{dQ_0}\right|\Delta\dot{Q}_0 + \left|\frac{d\eta}{dI}\right|\Delta I + \left|\frac{d\eta}{dA_\lambda}\right|\Delta A_\lambda \tag{S2}$$

$$\frac{d\eta}{da} = \frac{\sum mc_p}{I\left(1-10^{-A_\lambda}\right)} \tag{S3}$$

$$\frac{d\eta}{d\sum mc_p} = \frac{a}{I\left(1-10^{-A_\lambda}\right)} \tag{S4}$$

$$\frac{d\eta}{dQ_0} = -\frac{1}{I\left(1-10^{-A_\lambda}\right)} \tag{S5}$$

$$\frac{d\eta}{dI} = -\frac{a\sum mc_p - \dot{Q}_0}{\left(1-10^{-A_\lambda}\right)*I^2} \tag{S6}$$

$$\frac{d\eta}{dA_\lambda} = \frac{\left(a\sum mc_p - \dot{Q}_0\right)*10^{A_\lambda}*\log 10}{I*\left(10^{A_\lambda}-1\right)^2} \tag{S7}$$

The uncertainty of determination of the components of the equation was estimated based on the following considerations:

- Δa – standard error from fitting curve (Δa_{SE}) plus uncertainty of temperature determination (Equation S9); Equation S8 was transformed into a form:

$$a = \frac{b\left(T(t)-T_0\right)}{1-e^{-bt}} = \frac{bT}{1-e^{-bt}} \tag{S8}$$

$$\Delta a = \Delta a_{SE} + \left|\frac{da}{db}\right|\Delta b_{SE} + \left|\frac{da}{dT}\right|\Delta T + \left|\frac{da}{dt}\right|\Delta t \tag{S9}$$

  Here, also time of conducting experiment is included, meaning for t heading to infinity, the temperature difference should be established with greatest accuracy, but it is reasonable to conduct measurement by up to $4\tau = 4/b$, to minimize its impact.

$$\frac{da}{db} = \frac{T * e^{bt}\left(-1 + e^{bt} - bt\right)}{\left(e^{bt} - 1\right)^2} \cong T \tag{S10}$$

$$\frac{da}{dT} = \frac{b}{1 - e^{-bt}} \cong b \tag{S11}$$

$$\Delta t \cong 0 \tag{S12}$$

Due to the high temporal resolution (Δt = 0.03s), this part of equation could be also neglected.

$$\Delta a = \Delta a_{SE} + T * \Delta b_{SE} + b * \Delta T \tag{S13}$$

- $\Delta \sum mc_p$

$$\Delta \sum mc_p = m_{drop} * \Delta c_{p,drop} + c_{p,drop} * \Delta m_{drop} \tag{S14}$$

- $\Delta \dot{Q}_0$

$$\dot{Q}_0 = a_0 \sum mc_p \tag{S15}$$

$$\Delta \dot{Q}_0 = a_0 * \Delta \sum mc_p + \sum mc_p * \Delta a_0 \tag{S16}$$

- ΔI=0,03*I

  Measurement error of laser power is mostly governed by the optical power meter accuracy (3%).

- ΔA_λ – Absorbance measurement error

  From Lambert-Beer's law:

$$A_\lambda = \log \frac{I_w}{I_s} \tag{S17}$$

  where I_w and I_s are light transmission of water and sample,

$$\Delta A_\lambda = \frac{\Delta I_w}{I_w * \ln(10)} + \frac{\Delta I_s}{I_s * \ln(10)} = \frac{I_w * 0{,}03}{I_w * \ln(10)} + \frac{I_s * 0{,}03}{I_s * \ln(10)} = 0{,}026 \tag{S18}$$

Estimating the experimental parameters

- $Q_0 = 0, a_0 = 0, \Delta a_0 = \Delta a$

  We did not observe heating of water while irradiating with a 532 nm beam

- $\Delta m_{drop} = 6$ µg

  Estimated resolution of determining the mass of droplet by thermal imaging camera

visualization with improved edge determination (fitting the droplet edge data with a logistic curve)

- ΔT = 0.1 ^{0}C

  Estimated resolution of temperature differential measurements by thermal imaging camera with improved accuracy (artefacts eliminated by subtracting the background signal)

$$\Delta\eta = \left|\frac{d\eta}{da}\right|\Delta a + \left|\frac{d\eta}{d\sum mc_p}\right|\Delta\sum mc_p + \left|\frac{d\eta}{d\dot{Q}_0}\right|\Delta\dot{Q}_0 + \left|\frac{d\eta}{dI}\right|\Delta I + \left|\frac{d\eta}{dA_\lambda}\right|\Delta A_\lambda \cong 39.5\%$$

$$\Delta\eta = 1.1\% + 31.4\% + 1.0\% + 2.2\% + 3.8\% \cong 39.5\%$$

The analysis of errors, shows that the precision of the drop weight estimation based on its image has the greatest impact on the precision of the droplet volume determination. The reason for such a high value is the fact that the end of the tip with a diameter of 0.9 + 0.1 mm is used as a scale for determining the drop volume. The accuracy of the measurement can be therefore increased by using an independent, more accurate scale placed in the field of view.

Chen's model

Chen et al [1] firstly proposed an easy to reproduce experimental setup: a sample is in a spectrophotometric cuvette and the temperature of the sample is homogenous because of a use of a magnetic stirrer during experiments. Energy balance is the same as in equation (1), only the heat connected with solution was included in Q_L:

$$Q_L = I(1-\xi)(1-10^{-A_\lambda})\eta \qquad \text{(S19)}$$

In opposite to I from Roper's model[2], here I is a is the reflection-corrected laser power, ξ is a fraction of energy absorbed by surroundings (cuvette and water) and A_λ is extinction value, Energy dissipated from the system was modeled by a Taylor series of ΔT:

$$Q_{ext} = B\Delta T + C\Delta T^2 \tag{S20}$$

B and C were evaluated from cooling data (when $Q_L = 0$), similarly than in Roper's model,

$$\left(m_s c_{p,s} + m_c c_{p,c}\right)\frac{d\Delta T}{dt} = -B\Delta T - C(\Delta T)^2 \tag{S21}$$

Finally, light to heat conversion efficiency is:

$$\eta = \frac{B(T_{max} - T_{amb}) + C(T_{max} - T_{amb})^2 - I\xi}{I(1-\xi)\left(1 - 10^{-E_\lambda}\right)} \tag{S22}$$

Table S5. Comparison of existing physical models

	**Roper [2] (cuvette)**	**Richardson [3] (droplet)**	**Wang [4] (cuvette + stirring + "mass calibration")**
Standard setup description	Sample inside the sealed glass cell/cuvette	Droplet on the syringe	Stirred sample inside the sealed glass cell/cuvette
Efficiency equation	$\frac{\sum_i m_i C_{p,i}(T_{max} - T_{amb})}{\tau_c * I(1-10^{-A_\lambda})} - \frac{Q_0}{I(1-10^{-A_\lambda})}$	$\frac{m_w C_{p,w}(T_{max} - T_{amb})}{\tau_c * I(1-10^{-A_\lambda})}$	$\frac{a \sum_i m_i C_{p,i}}{I(1-10^{-A_\lambda})}$
Heat transfer processes included in model	Convection and conduction (no geometry included)	Convection of a sample	Convection (spontaneous) and conduction
Most important physical processes in the setup	Sample – convection<br>Glass – conduction<br>Glass-air – convection (eliminated when measured in a vacuum)	Sample – convection<br>Syringe – conduction	Sample – forced convection<br>Glass – conduction<br>Glass-air - convection
Additional problems	Geometry of a system is not included in the model	Evaporation of a sample	Stirring not included in the model

(1) Chen, H.; Shao, L.; Ming, T.; Sun, Z.; Zhao, C.; Yang, B.; Wang, J. Understanding the Photothermal Conversion Efficiency of Gold Nanocrystals. *Small* **2010**, *6* (20), 2272–2280. https://doi.org/10.1002/smll.201001109.

(2) Roper, D. K.; Ahn, W.; Hoepfner, M. Microscale Heat Transfer Transduced by Surface Plasmon Resonant Gold Nanoparticles. *J. Phys. Chem. C* **2007**, *111* (9), 3636–3641. https://doi.org/10.1021/jp064341w.

(3) Richardson, H. H.; Carlson, M. T.; Tandler, P. J.; Hernandez, P.; Govorov, A. O. Experimental and Theoretical Studies of Light-to-Heat Conversion and Collective Heating Effects in Metal Nanoparticle Solutions. *Nano Lett.* **2009**, *9* (3), 1139–1146. https://doi.org/10.1021/nl8036905.

(4) Wang, X.; Li, G.; Ding, Y.; Sun, S. Understanding the Photothermal Effect of Gold Nanostars and Nanorods for Biomedical Applications. *RSC Adv.* **2014**, *4* (57), 30375–30383. https://doi.org/10.1039/C4RA02978J.
